# Lung carcinoma with diffuse cystic lesions misdiagnosed as pulmonary langerhans cell histocytosis: a case report

**DOI:** 10.1186/s12890-020-1066-5

**Published:** 2020-02-04

**Authors:** Xianhua Gui, Jingjing Ding, Yan Li, Min Yu, Tingting Chen, Mei Huang, Yonglong Xiao

**Affiliations:** 1 Department of Respiratory and Critical Care Medicine, The Affiliated Drum Tower Hospital of Nanjing University Medical School, No. 321 Zhongshan Road, Nanjing, 210008 Jiangsu People’s Republic of China; 2 Department of Pathology, The Affiliated Drum Tower Hospital of Nanjing University Medical School, No. 321 Zhongshan Road, Nanjing, 210008 Jiangsu People’s Republic of China

**Keywords:** Diffuse cystic lesion, Chest computed tomography, Lung adenocarcinoma

## Abstract

**Background:**

Cystic airspace is an uncommon imaging manifestation involved in non-small lung cancer (NSCLC). Diffuse cystic lesion is even rarer as pulmonary manifestation of NSCLC. In the present study, we reported a rare case of NSCLC associated with progressive diffusion of cystic lesions misdiagnosed as Pulmonary langerhans cell histocytosis (PLCH), finally diagnosed by transbronchial cryobiopsy (TBCB).

**Case presentation:**

A 52-year-old woman was admitted to our hospital due to cough and dyspnea. High-resolution computed tomography (HRCT) presented diffuse cystic shadow mostly, concomitantly with nodular densities in bilateral lungs. A lung biopsy revealed poorly differentiated adenocarcinoma with vascular tumor emboli. The epidermal growth factor receptor (EGFR) mutation on exon 18 (G719X, G719) was detected by mutation test. The patient received treatment of tyrosine kinase inhibitor (afatinib).

**Conclusions:**

Diffuse cystic lesion can be a rare manifestation of lung cancer. It was important to improve the recognition of diffuse cystic lung diseases to avoid misdiagnosis.

## Background

Diffuse cystic lung diseases (DCLDs) are a group of pathophysiologically heterogenous processes characterized by the formation of multiple thin-walled cystic lesions in the lung parenchyma [1]. DCLDs are rarely caused by a malignant process, which are secondary to metastases from adenocarcinomas of the gastrointestinal tract [2, 3] and sarcomas of various cell types [4, 5]. In the present study, we described the clinical characteristics of a DCLD patient associated with lung cancer diagnosed by transbronchial cryobiopsy (TBCB) in our hospital. Our findings provided valuable insights into the early identification of this type of lung cancer.

## Case presentation

**Fig. 1:**
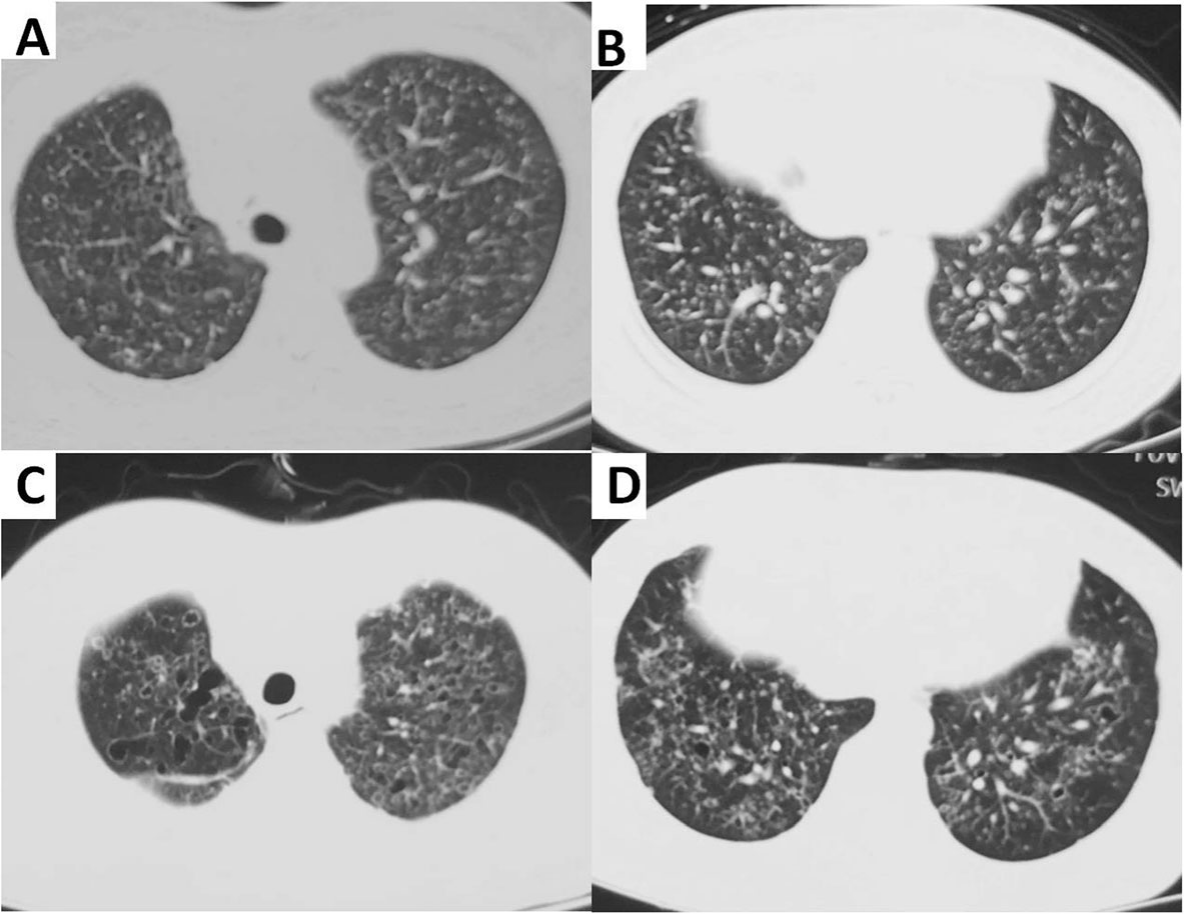
Computed tomography (CT) scan of the thorax. **a**, **b** CT of the thorax on February 2018 showed diffuse nodules and cysts distributing in bilateral lung. **c**, **d** Repeated Chest CT on August 2018 revealed enlarged, widespread, disseminated thin-walled cysts with different shape

A 52-year-old woman was referred to our hospital with a 1-year history of progressive breathlessness and productive cough with white sputum. The patient had no occupational exposure and had a ten-year smoking history. Computed tomography (CT) showed multiple cysts and nodules (Fig. 1 a and b). Pulmonary langerhans cell histocytosis (PLCH) was considered basing on the imaging manifestation and smoking history without evidence of histopathology due to limited medical care, and treated with giving up smoking. After 6 months, repeated chest CT scan revealed rapid progress, exhibiting disseminated thin-walled cystic lesions in bilateral lungs (Fig. 1 c and d). Finally, the patient was transferred to our hospital with progressive breathlessness and aggravated cough.

Physical examination on admission included body temperature 36.3 °C, the blood pressure 115/74 mmHg, respiratory frequence 19 times per minute, heart rate 80 times per minute and auscultation of her lung revealed decreased breath sounds. The oxygen saturation (SpO2) was 93% in ambient air.

Significant laboratory values were as follows: normal autoimmune markers, normal anti-neutrophil cytoplasmic antibody level, no evidence of infection in sputum culture, an increased serum white cell count of 11.6 × 10^9^/L (4–10 × 10^9^/L). A severely mixed ventilatory defect was detected by pulmonary function test, showing a forced vital capacity (FVC) of 1.37 L (47.6% of predicted value), a forced expiratory volume in 1 s (FEV1) of 1.00 L (42.6% of predicted value) and severe reduction of diffusion capacity (DLCO) of 1.94 L (27.4% of predicted value). Echocardiogram showed mild-to-moderate pulmonary hypertension (48 mmHg).

**Fig. 2:**
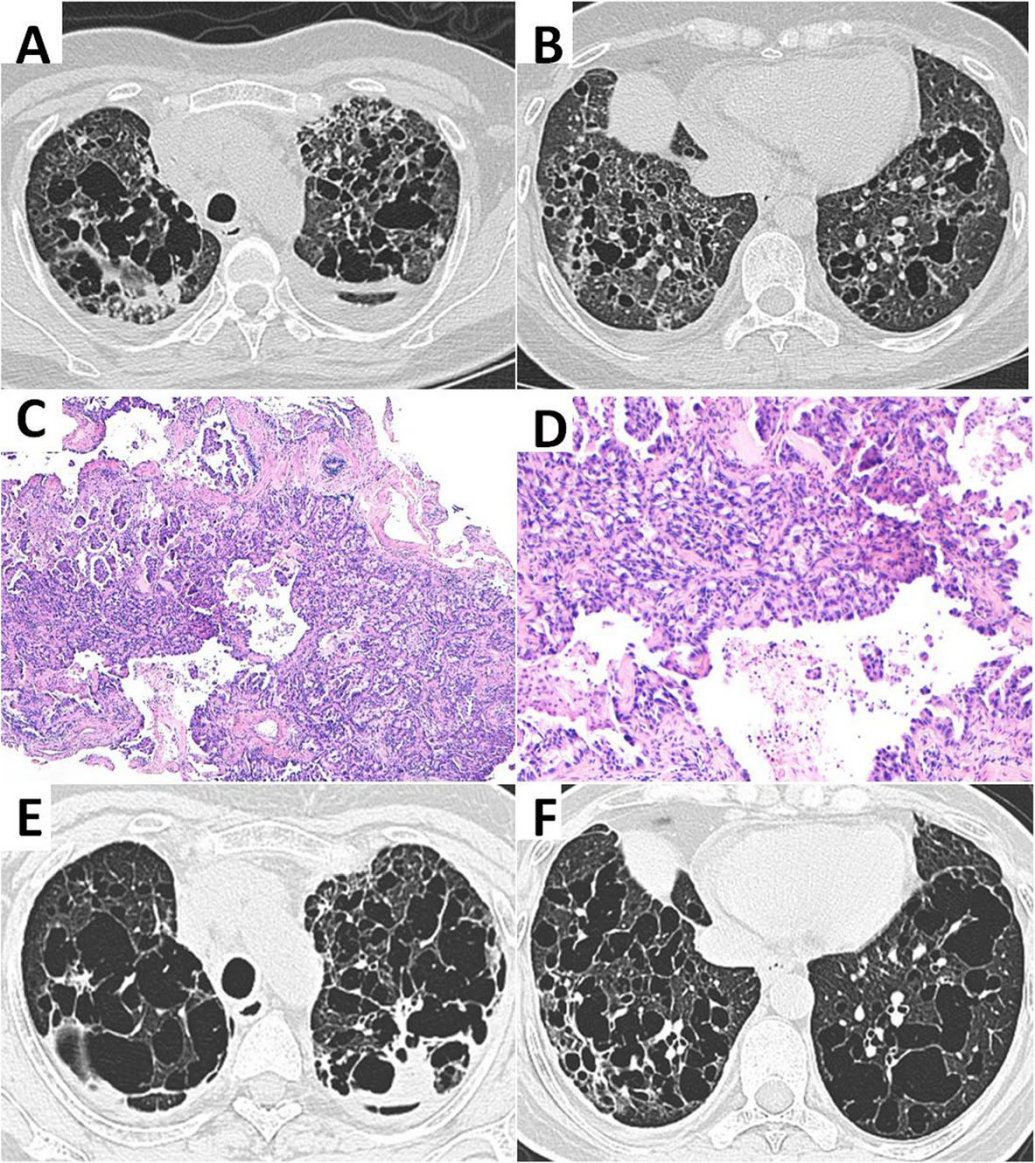
Chest CT on 2019 and microscopic examination of specimens. **a**, **b** Chest CT on March 2019 showed rapid progress, disseminated thin-walled cystic lesions and nodules in bilateral lungs. **c**, **d** The pathology revealed poorly differentiated adenocarcinoma and vascular tumor emboli. **e**, **f** Pulmonary nodules diminished, and the cysts fused bizarrely shaped cysts after 1 month of afatinib therapy

Repeated HRCT revealed obvious deterioration with diffuse pulmonary cystic lesions, pericardial effusion and bilateral pleural effusion (Fig. 2 a and b). Bronchoalveolar lavage fluid cytology indicated 5% neutrophils, 5% lymphocytes, 55% histocytes, 10% ciliated cells and 5% cancer cells. TBCB showed poorly differentiated adenocarcinoma and vascular tumor emboli (Fig. 2 c and d). Immunohistochemical analysis revealed the lung primary site [cytokeratin (CK) 7 and thyroid transcription factor 1 (TTF1) positive]. Abnormal carcino-embryonic antigen (CEA) level of 50.93 ng/mL (0–10 ng/mL) and an increased neuron-specific enolase (NSE) level of 4.26 ng/mL (0–3.3 ng/mL) were observed. Magnetic resonance imaging (MRI) of the brain suggested possible metastatic tumor in the left frontal lobe. Bone scintigraphic imaging showed multiple bone metastases. Therefore, her clinical stage was T4N0M1c stage IV, and epidermal growth factor receptor (EGFR) mutation test was positive with exon 18 (G719X, G719). She underwent afatinib therapy. The next follow-up after 1 month demonstrated significant improvement, and the patient was alive (Fig. 2 e and f).

## Discussion and conclusions

DCLD is a set of independent lung diseases, including pulmonary metastases, pulmonary langerhans cell histocytosis (PLCH), lymphangioleiomyomatosis (LAM), Birt-Hogg Dube (BHD) syndrome and so on [1, 6]. To date, studies on solitary cystic lung cancer have reported that 3.7% lung cancer cases are associated with cystic airspaces [7]. However, diffuse cysts are even rarer as pulmonary appearance of non-small lung cancer (NSCLC). To date, DCLDs reported in literature are case reports with adenocarcinoma as the pathologic types of diffuse cystic lung cancer [8, 9]. It has a unique imaging manifestation different from common lung cancer, which may be a potential cause of misdiagnosis.

The clinical presentation of NSCLC with diffuse cystic lesions is remarkably different from common lung cancer. The patients primarily present with a cough and dyspnea secondary to diffuse infiltration of tumor cells [8, 9]. In our patient, this was reflected by a mixed ventilatory defect with seriously reduced diffusion capacity of carbon monoxide in pulmonary function test. Affected patients are more likely to develop to respiratory failure.

The radiographic definition of diffuse cystic lesion of the lung is characterized by a thin-walled (< 2 mm), air-filled, spherical lucency in the lung parenchyma. The mechanism underlying the formation of parenchymal cysts in NSCLC remains uncertain. Some scholars have speculated that accumulation of tumor cells in terminal and respiratory bronchioles forms a one-way valve, resulting in excessive peripheral ventilation [6, 10, 11]. It has also been proposed that ischemic necrosis of terminal bronchioles and alveoli is induced by infiltration of small vessels and capillaries, leading to alveolar ischemic dilation to form cysts [12]. Our results also suggested an alveolar ischemic dilation mechanism, where the tumor cells infiltrated in vascular system, resulting in alveolar ischemic dilation.

There are a broad set of diseases based on the special imaging manifestation. Accompanying features with invaluable clues can help us distinguish different types of DCLDs, including commonly observed PLCH and LAM. PLCH usually occurs in smokers at 20 ~ 40 years old, who had a long history of smoking or exposure to second-hand smoke [13]. Centrilobular nodules and bizarrely shaped cysts can appear mainly in upper and middle lung lobe on chest HRCT. Early histopathology reveals bronchiolocentric nodules (accumulation of langerhans cells, eosinophils, lymphocytes and fibroblasts). In later stages, cysts can form because of enlarged airspace and cavitary nodules. Pulmonary langerhans cells positively expressing CD1a and S-100 can help to diagnose such disease [14]. NSCLC associated with diffuse cystic lesions have the similar imaging manifestation to PLCH, including nodules and cysts, making it difficult to distinguish. As a rare cystic disease, LAM is usually characterized by pulmonary infiltration of smooth muscle cells harboring tuberous sclerosis genes with growth-activating mutations, and such situation often occurs in women of childbearing age [15, 16]. The chest HRCT presents diffuse distribution of uniform-sized thin-walled cysts, which is often accompanied with renal angiomyolipomas on abdominal CT [17]. Thus, similar imaging manifestations make it difficult to distinguish LAM from other cystic lung disease. The final diagnoses depend on the pathology of lung tissue.

Collectively, diffuse thin-walled cysts in chest-CT are rare imaging manifestations of lung cancer, which is often ignored and misdiagnosed as other cystic lung diseases due to similar imaging findings. Early recognition of this infrequent radiologic feature of lung cancer and prompt therapy are important.

## Data Availability

The datasets used and/or analyzed during the current study available from the corresponding author on reasonable request.

## Abbreviations

CEA: Carcino-embryonic antigen
DCLDs: Diffuse cystic lung diseases
DLCO: Diffusion capacity
FEV1: Forced expiratory volume in 1 s
FVC: Forced vital capacity
HRCT: High-resolution computed tomography
LAM: Lymphangioleiomyomatosis
MRI: Magnetic resonance imaging
NSCLC: Non-small lung cancer
NSE: Neuron-specific enolase
PLCH: Pulmonary langerhans cell histocytosis
SpO_2_: Oxygen saturation
TBCB: Transbronchial cryobiopsy
